# Hydrogels in wearable neural interfaces

**DOI:** 10.1007/s44258-024-00040-4

**Published:** 2024-12-09

**Authors:** Mengmeng Yao, Ju-Chun Hsieh, Kai Wing Kevin Tang, Huiliang Wang

**Affiliations:** Department of Biomedical Engineering, The University of Texas at Austin, Austin, TX 78712 USA

**Keywords:** Hydrogels, Wearable neural interface, Compatibility, Impedance, Conductivity, Adhesiveness, Neural recording, Neurostimulation

## Abstract

The integration of wearable neural interfaces (WNIs) with the human nervous system has marked a significant progression, enabling progress in medical treatments and technology integration. Hydrogels, distinguished by their high-water content, low interfacial impedance, conductivity, adhesion, and mechanical compliance, effectively address the rigidity and biocompatibility issues common in traditional materials. This review highlights their important parameters—biocompatibility, interfacial impedance, conductivity, and adhesiveness—that are integral to their function in WNIs. The applications of hydrogels in wearable neural recording and neurostimulation are discussed in detail. Finally, the opportunities and challenges faced by hydrogels for WNIs are summarized and prospected. This review aims to offer a thorough examination of hydrogel technology’s present landscape and to encourage continued exploration and innovation. As developments progress, hydrogels are poised to revolutionize wearable neural interfaces, offering significant enhancements in healthcare and technological applications.

**Graphical Abstract:**

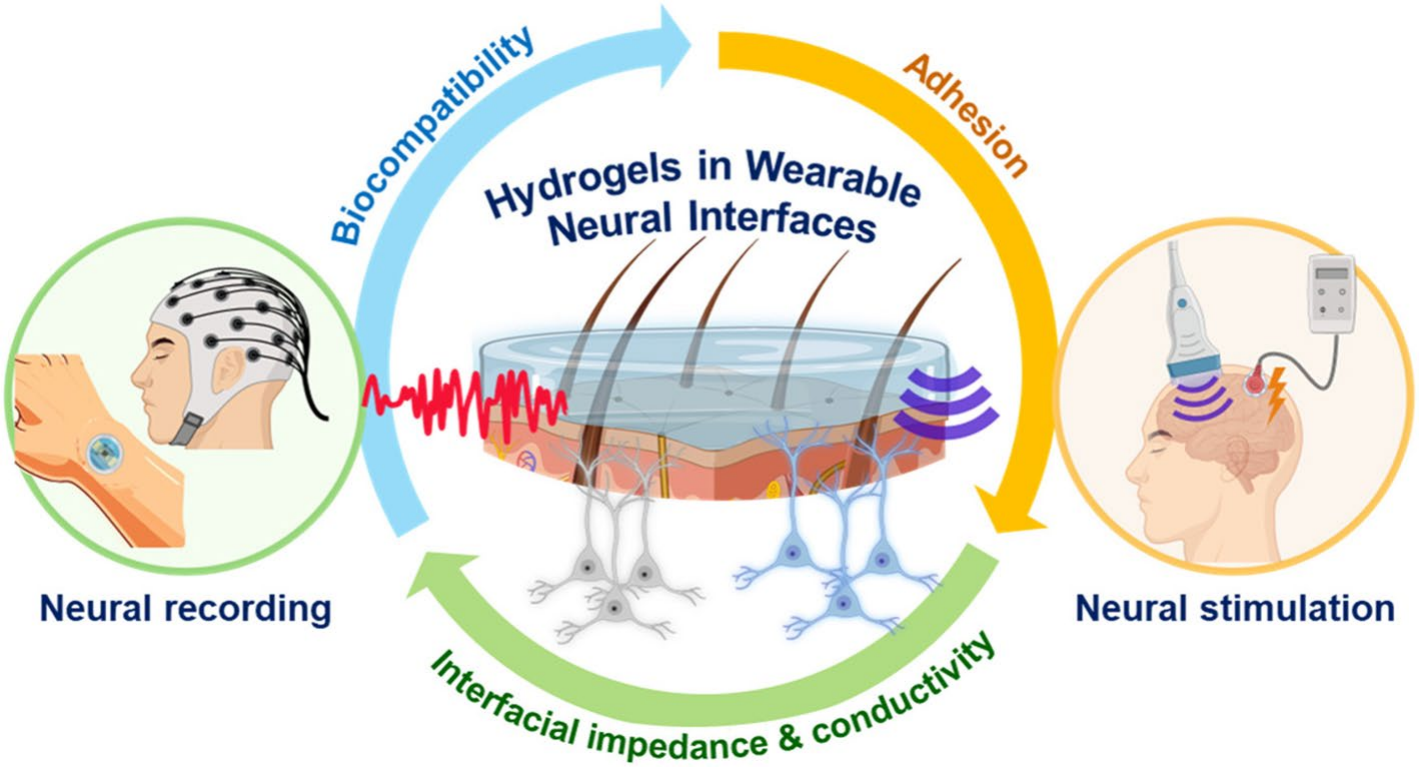

## Highlights

• The biocompatibility, interfacial impedance and conductivity, and adhesion of hydrogel are critical to the functionality of hydrogels in wearable neural interfaces.

• Hydrogels can effectively support the requirements for wearable neural recording and neurostimulation applications.

• This review discusses the opportunities and challenges of using hydrogels in wearable neural interfaces.

## Introduction

In the rapidly evolving field of biomedical engineering, the development of neural interfaces represents a significant leap forward in harnessing the power of the human nervous system for therapeutic and enhancement purposes [1–6]. These interfaces, which establish direct pathways for communicating between the neural system and external devices, are increasingly pivotal in both medical treatments and the integration of technology into human sensory and motor systems [7–14]. Among these, wearable neural interfaces (WNIs) have emerged as a particularly innovative subset, offering the promise of seamless integration and real-time interaction with neural circuits through non-invasive or minimally invasive means [1, 13, 15–19].

However, integrating WNIs with human skin presents unique challenges, primarily related to the biocompatibility, durability, and functionality of the materials used [20–22]. Traditional materials often face issues such as rigidity [23–28], which limits conformity to the skin, and potential irritative or allergic reactions, which can impair long-term usability and comfort [15, 29, 30]. Moreover, the mechanical mismatch between stiff electronic components and the soft, dynamic nature of human skin can lead to inefficient signal transmission, highlighting the need for more adaptable and skin-friendly materials [20, 31–33].

Hydrogels, which are networks of hydrophilic polymer chains that can hold a large amount of water, making them highly flexible and similar to natural tissue [9, 34–37]. This compatibility enables a much gentler interface with human skin, enhancing comfort and reducing the risk of adverse reactions [38–41]. Furthermore, their porous structure allows for the transmission of bio-signals and the incorporation of sensors and electrodes, making them ideal for continuous monitoring and interaction with the neural system [42–45]. The critical role of hydrogels in WNIs stems not only from their physical and chemical versatility but also from their potential to revolutionize the design and functionality of wearable devices [7, 38, 46–48]. By addressing the core challenges associated with traditional materials, hydrogels pave the way for more effective, durable, and user-friendly neural interfaces.

This review aims to provide a comprehensive overview of the current state of hydrogel technology for WNIs and inspire future research and development. Furthermore, we explore the critical properties and applications of hydrogels in WNIs, highlighting their pivotal role in enhancing device functionality and user comfort. With ongoing advancements, hydrogels are poised to revolutionize WNIs, promising significant improvements in both healthcare and technology.

## Critical parameters of hydrogels in wearable neural interfaces

Hydrogels have emerged as frontrunners in the development of WNIs due to their unique properties, which closely align with the requirements for prolonged and effective human-device interaction [15, 49–51]. These polymeric networks, capable of holding large amounts of water, provide a soft, compliant, yet robust medium that can conform to complex body contours while maintaining functionality [34, 52]. In this section, we focus on four pivotal attributes of hydrogels—biocompatibility, interfacial impedance, conductivity, and adhesiveness—which are essential for their successful integration into long-term wearable neural devices. We explore each of these properties, discussing how they contribute to the overall performance of WNIs and the challenges that remain in optimizing their application.

### Biocompatibility

Biocompatibility is paramount for any material intended in contact with the human body. Hydrogels are particularly valued in medical applications for their excellent biocompatibility [34, 53]. This attribute stems from their hydrophilic nature, which allows them to integrate well with biological environments, minimizing irritation or allergic response while promoting skin interface integration [37, 42, 54]. The discussion will cover various hydrogel formulations that have been engineered to optimize biocompatibility, focusing on their chemical composition, physical structure, and the resultant biological interactions.

**Fig. 1:**
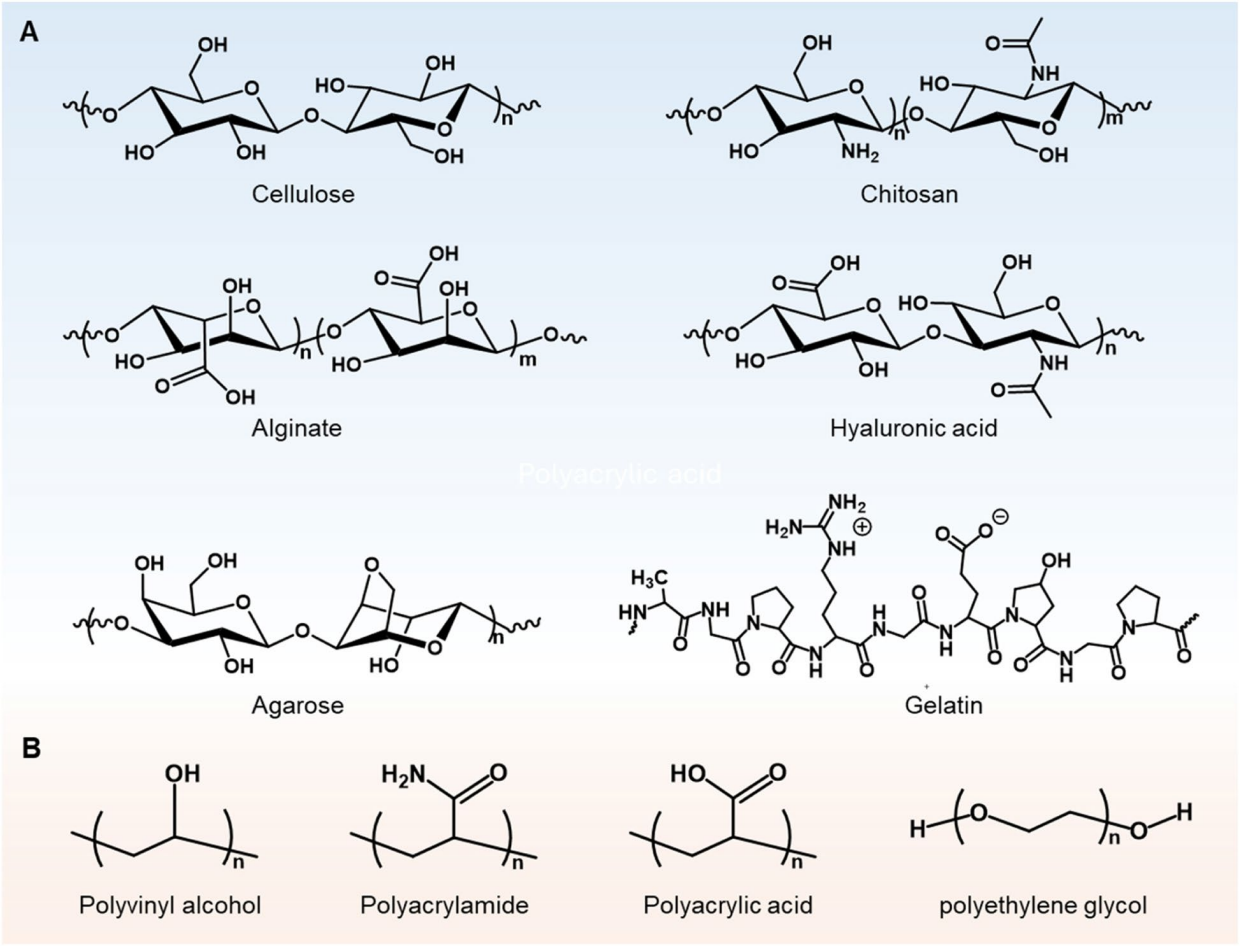
The chemical structural of representative (**A**) natural polymer materials and (**B**) synthetic polymer materials commonly used in hydrogels for neural wearable interfaces

Hydrogels are increasingly recognized as essential materials in the development of wearable neuro devices due to their biocompatibility, flexibility, and close interfacing capabilities with biological tissues. These hydrogels are predominantly made from hydrophilic polymer networks that can be natural, synthetic, or a hybrid combination. Natural polymers such as alginate [1, 35, 55–58], chitosan [59–62], and proteins (silk fibroin or gelatin) [63–69] are preferred for their inherent biocompatibility and bioactive properties, while synthetic options like polyethylene glycol [70–72], polyacrylamide [35, 42, 55, 65, 73–76], polyvinyl alcohol [77–81], polyacrylic acid [40, 82–84], and poly (2-Acrylamido-2-methylpropane sulfonic acid) [52, 85, 86] offer enhanced control over the hydrogel’s mechanical characteristics. The structural formula of natural polymer materials and synthetic polymer materials commonly used in hydrogels are shown in Fig. 1A and B. The method of chemical crosslinking in these hydrogels is crucial, as it affects both the physical attributes and the biological response of the material. Chemical crosslinkers, such as *N, N*-Methylenebis(acrylamide), or physical interactions like ionic bonding, are employed depending on the desired properties. The engineered physical structure of hydrogels, including their porosity, mesh size, and stiffness, is designed to mimic the natural extracellular matrix, promoting better integration with the skin interface [55, 87, 88]. The evaluation of biocompatibility for hydrogels in WNIs primarily focuses on the extent of interaction between the material and the skin. Key factors considered in this evaluation include cytotoxicity and skin irritation/sensitization [49]. Although hydrogels in wearable devices are not implanted, they remain in prolonged contact with the skin. Cytotoxicity tests, such as MTT or Live/Dead assays, are conducted to ensure that the hydrogel does not release harmful substances that could damage skin cells [89, 90]. Additionally, because of extended skin contact, the potential for skin irritation and sensitization is a critical concern. Patch tests or similar assessments are employed to determine whether the hydrogel induces irritation, redness, or allergic reactions after long-term wear. To ensure safety and comfort during extended use, the material must minimize the risk of skin inflammation or allergic responses. The biocompatibility of hydrogels in WNIs is closely related to their structure, including crosslinking density and surface chemistry [91]. Crosslinking density plays a crucial role in balancing flexibility and stability. Lower crosslinking density provides a softer material that conforms better to the skin, improving comfort and reducing irritation over long durations [92]. However, excessively low crosslinking density may compromise mechanical strength, affecting the durability needed for daily wear. Surface chemical modifications, such as the introduction of hydrophilic or antimicrobial groups, minimize bacterial colonization and protein fouling, enhancing biocompatibility in WNIs [93, 94]. In conclusion, the structural properties of hydrogels, including their crosslinking, surface modifications, and porosity, play a critical role in ensuring biocompatibility in WNIs designed for non-invasive and long-term use.

### Interfacial impedance

**Fig. 2:**
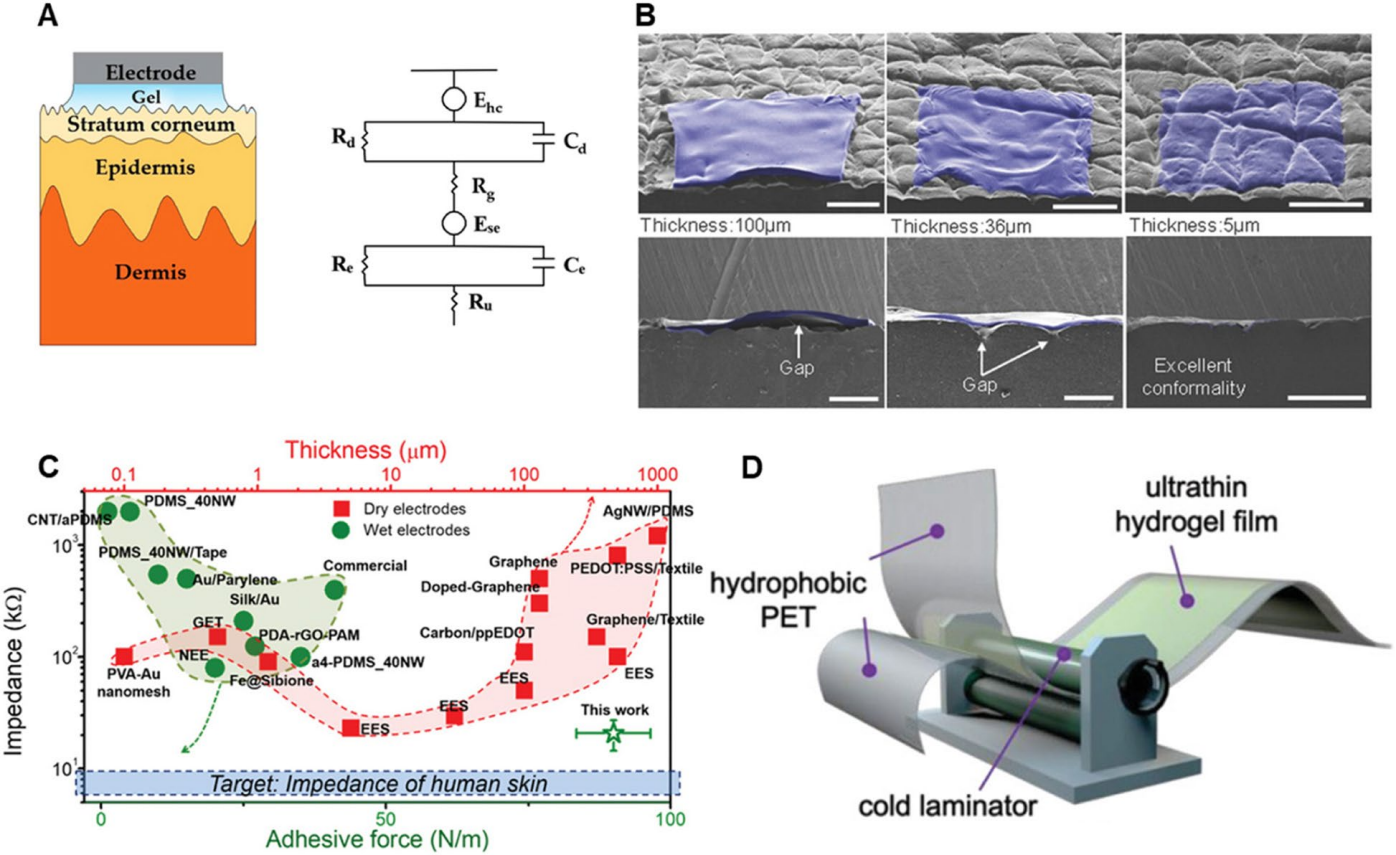
**A** Schematic and electrical equivalent circuit model of hydrogel electrode [106]. **B** Angled and cross-sectional SEM images showing degree of conformal contact between a silicone replica of the surface of the skin (grey) and various thicknesses of elastomer membrane substrates (blue) for epidermal electronic systems [102]. **C** The interfacial impedance and adhesion/thickness of different wet/dry electrodes. The green circles represent the wet electrodes, the red squares are the dry electrodes [104]. **D** Schematic of the cold-lamination method to produce large-area ultrathin hydrogel films [105]

The impedance of hydrogel electrodes in WNIs refers to the resistance these electrodes offer to the flow of alternating current across various frequencies during neural recording or stimulation (Fig. 2A) [95, 96]. In devices like electroencephalogram (EEG) monitoring systems, the impedance of the electrode directly affects the signal quality. Higher impedance not only weakens the biological signals captured by the electrode, but also increases the interference of external noise, resulting in a decrease in signal quality. Therefore, studying and optimizing the impedance performance of hydrogel electrodes is crucial to improving the reliability and accuracy of WNIs [97]. First, the geometry of the electrode is one of the key factors affecting impedance [98, 99]. Generally, the thickness and surface area of ​​the electrode show a direct correlation with the impedance. Thicker electrodes increase resistance due to longer current transmission paths, while smaller surface areas reduce the contact area between the electrode and the skin, resulting in higher impedance [100]. Electrodes with extensive contact areas typically show reduced impedance. This enhancement is evident in the electrode’s overall dimensions and surface texture, where rough textures yield a larger surface area. For example, Yun et al. [101] increased the surface area of a flexible polyimide substrate by 1.54 times using gold nanoparticle electro-deposition. This modification resulted in electrodes with significantly higher signal-to-noise ratios (SNR) in electromyography recordings compared to standard commercial Ag/AgCl electrodes. In addition to the geometry of the electrode, the interface impedance between the electrode and the skin or tissue is another important factor affecting the total impedance. As reported by Rogers et al., the total energy of interface contact (*U*_*interface*_) is composed of the bending energy of the electrode (*U*_*bending*_) (mainly dependent on the thickness of the electrode), the elastic energy of the skin (*U*_*skin*_) and the adhesion energy of the contact (*U*_*adhesion*_), which is expressed as follows [102, 103]: ${U}_{interface}={U}_{bending}+{U}_{skin}+{U}_{adhesion}$. To minimize the interface gap to comply with the low impedance of the skin, reducing thickness and improving adhesion (Fig. 2B and C), which we will summarize in Sect. " Conductivity", are effective strategies for bothe dry electrodes and wet electrodes [104]. However, it is difficult to prepare ultrathin hydrogel films with controllable thickness and uniformity using traditional casting or spin-coating methods. Recently, Cheng et al. [105] developed a cold lamination method that enables large-area production of ultrathin hydrogel films with a thickness of 10 µm with controllable uniformity (Fig. 2D). These hydrogel films gently cover the texture and fine details of the skin’s surface without creating any air gaps. They further verified the physical coupling of hydrogel to skin glyphic patterns, following the well-established analytical mechanics model by Rogers and co-workers. As thickness decreased, the hydrogel exhibited significantly improved compliance, showing that confirmation of wrinkles with narrower structure and wider depth range. Additionally, Table 1 also summarizes several strategies to enhance the interfacial impedance of hydrogels and how each strategy can interact with others. This table highlights the interconnectedness of the strategies, demonstrating how they collectively contribute to optimizing the interfacial impedance of hydrogel for better performance in WNIs.

**Table 1:** Interface impedance optimization strategies and comparative matrix of hydrogels for wearable neural interfaces

Strategy Category	Description	Comparative Insights
Hydrogel Formulation [55, 104, 107–109]	Adjusting polymer composition and cross-linking density Incorporation of conductive nanoparticles	Works with hydration management to maintain polymer conductivity
Hydrogel Morphology [50, 110, 111]	Adjusting pore structure to enhance ion transport Modifying electrode thickness or increasing surface area	Enhances interface improvements by integration with the skin and adhesive layers
Skin Preparation [50, 112]	Using mild abrasives or ethanol to disrupt the stratum corneum	Improves electrode geometry effectiveness by ensuring skin–electrode conformity
Interface Improvements [104, 113, 114]	Creating adhesive layers to enhance skin attachment	Supports electrode geometry by optimizing the contact quality
Electrode Geometry [115, 116]	Customizing the shape and arrangement of electrodes to match the anatomical features of the target area	Affects all other strategies by determining the structural context in which they operate

### Conductivity

**Fig. 3:**
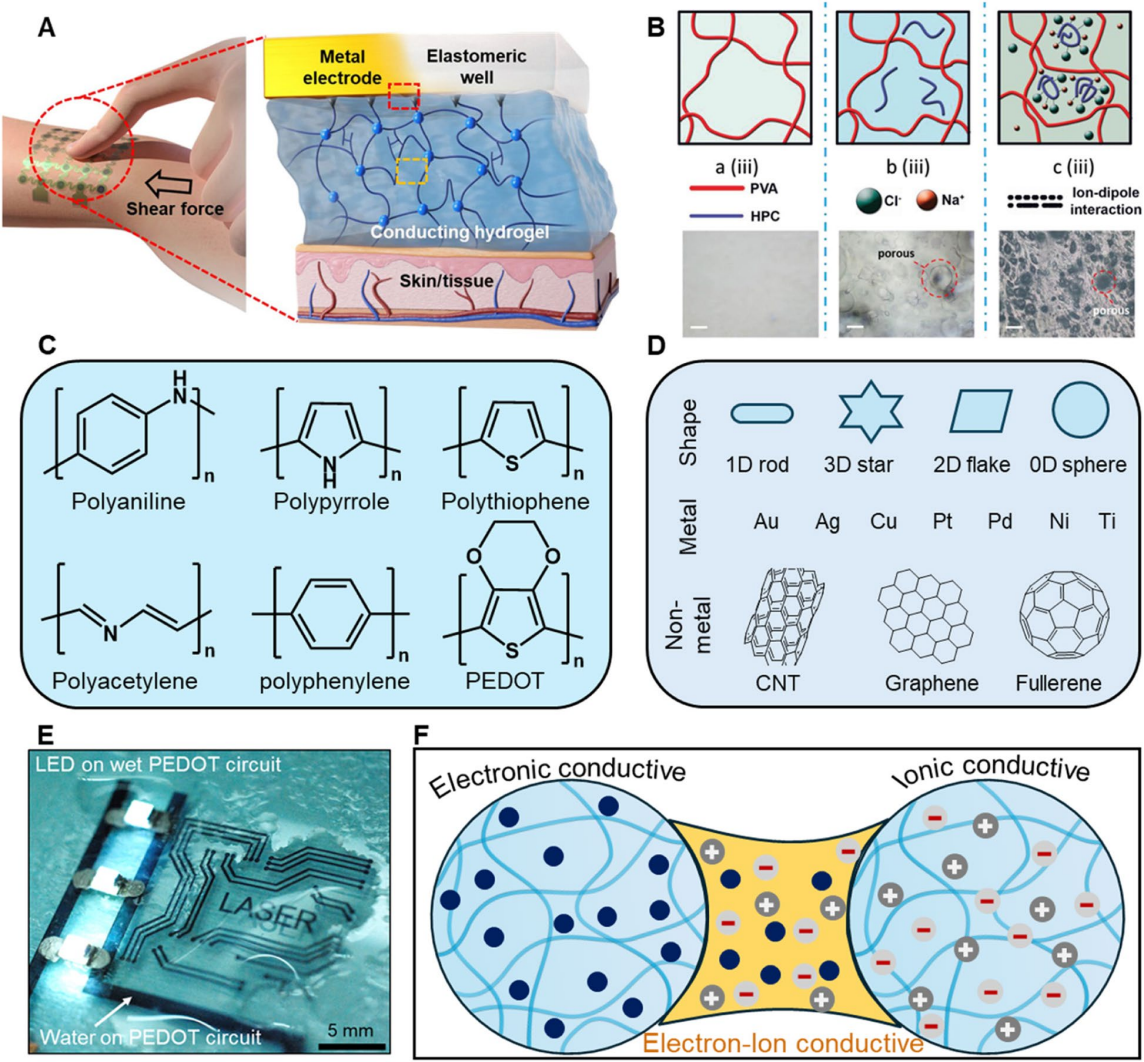
**A** Reliable and monolithic adhesion between the conductive hydrogel and the stretchable device and skin interface [95]. **B** Schematic of the formation of hydroxypropyl cellulose (HPC)/polyvinyl alcohol (PVA) ionic conductive hydrogel (soaked in 5 M NaCl solution), where Na^+^ and Cl^−^ ions were attracted by HPC fibers through ion–dipole interaction and pores in HPC/PVA ionic conductive hydrogel were retained. The scale bar of microscope images is 100 µm [77]. **C** Chemical structures of representative conductive polymers and (**D**) other representative metal and non-metal conductive components. **E** Micropatterned Poly(3,4-ethylenedioxythiophene): poly(styrenesulfonic acid) (PEDOT:PSS) hydrogel circuit turning on three LEDs in the hydrated state for neural recording [147]. **F** The dual capability of Electron–Ion conductive hydrogel is crucial for devices that process biological data and interface with electronic systems

The conductivity of hydrogels is another critical feature of wearable neural devices applied to human skin surfaces [77, 117]. It enables the efficient transmission of electrical signals between the device and the skin, mimicking or interfacing with the body’s neural networks (Fig. 3A) [118–120]. Ionic conductivity in hydrogels is facilitated by the movement of ions through the water-swollen network of the polymer and can be influenced by several factors [77, 121, 122]. Typically, higher water content enhances the mobility of ions within the hydrogel, thereby increasing conductivity. In addition, the choice of monomers and the crosslinking density can affect the pore size and structure of the hydrogel, which in turn influences ion transport pathways. Incorporating salts or other electrolytes into the hydrogel can increase the availability of free ions (Li^+^, K^+^, Na^+^, Cl^−^), thus improving conductivity (Fig. 3B) [123–128]. Water loss significantly impacts on the ionic conductivity of hydrogels. Several strategies can be employed to mitigate water evaporation and maintain conductivity. Adding moisturizing additives such as glycerol and polyethylene glycol helps attract and retain water within the hydrogel matrix, slowing the dehydration process and preserving the material’s ionic conductivity [129]. Additionally, applying moisture barrier coatings made of biocompatible polymers, like polyurethane or silicone, can limit water evaporation while maintaining the breathability of the material, ensuring that the hydrogel functions effectively over extended periods [130]. A layered hydrogel design, where a conductive inner layer is protected by an outer layer with enhanced water retention properties, can form a dual barrier to prevent water loss and sustains long-term ionic conductivity [131]. Unlike ionic conductivity, electronic conductivity in hydrogels involves the movement of electrons or holes and is less common in hydrogel materials due to their generally non-conductive nature [95]. However, electronic conductivity can be engineered into hydrogels by incorporating conductive materials such as metal nanoparticles, carbon nanotubes, or conductive polymers (Fig. 3C and D) [132–143]. Electronic conductivity is particularly important for the rapid and efficient transfer of electronic signals, enabling real-time sensing and response capabilities in wearable devices. For example, in a neural interface, electronically conductive hydrogels can help record and stimulate neural signals with high precision and minimal signal loss (Fig. 3E). Notably, the physiological environments display inherent ionic conductivity in the range of 0.3 to 0.7 S/m, largely due to the high-water content in biological tissues and the abundance of ions from salts and charged proteins present in these tissues [34]. Hence, the conductivity of hydrogel interfaces should be substantially higher than that of the surrounding tissue to ensure the quality of recorded signals. For WNIs, the ionic conductivity of hydrogels typically ranges from 10^–1^ to 10 S/m, depending on factors such as water content, ion concentration, and crosslinking density. The electronic conductivity of hydrogels can vary significantly depending on the type and concentration of the conductive materials used, with typical values ranging from 10^–1^ to 10^3^ S/m ^9^. In wearable neural devices, ionic conductivity ensures compatibility with the body’s natural ionic signals, while electronic conductivity enables the device to function efficiently with electronic components [144–146]. This dual capability is crucial for devices that must process biological data and interface with electronic systems, thereby boosting the utility and scope of wearable tech in medical diagnostics and treatment (Fig. 3F). Notably, ensuring biocompatibility is critical, as adding conductive materials to enhance functionality can compromise the hydrogel’s biocompatibility and mechanical properties. Moreover, hydrogels should maintain their conductivity and structural integrity over time due to their being susceptible to degradation from environmental factors such as temperature, humidity, and mechanical stress.

### Adhesion

**Fig. 4:**
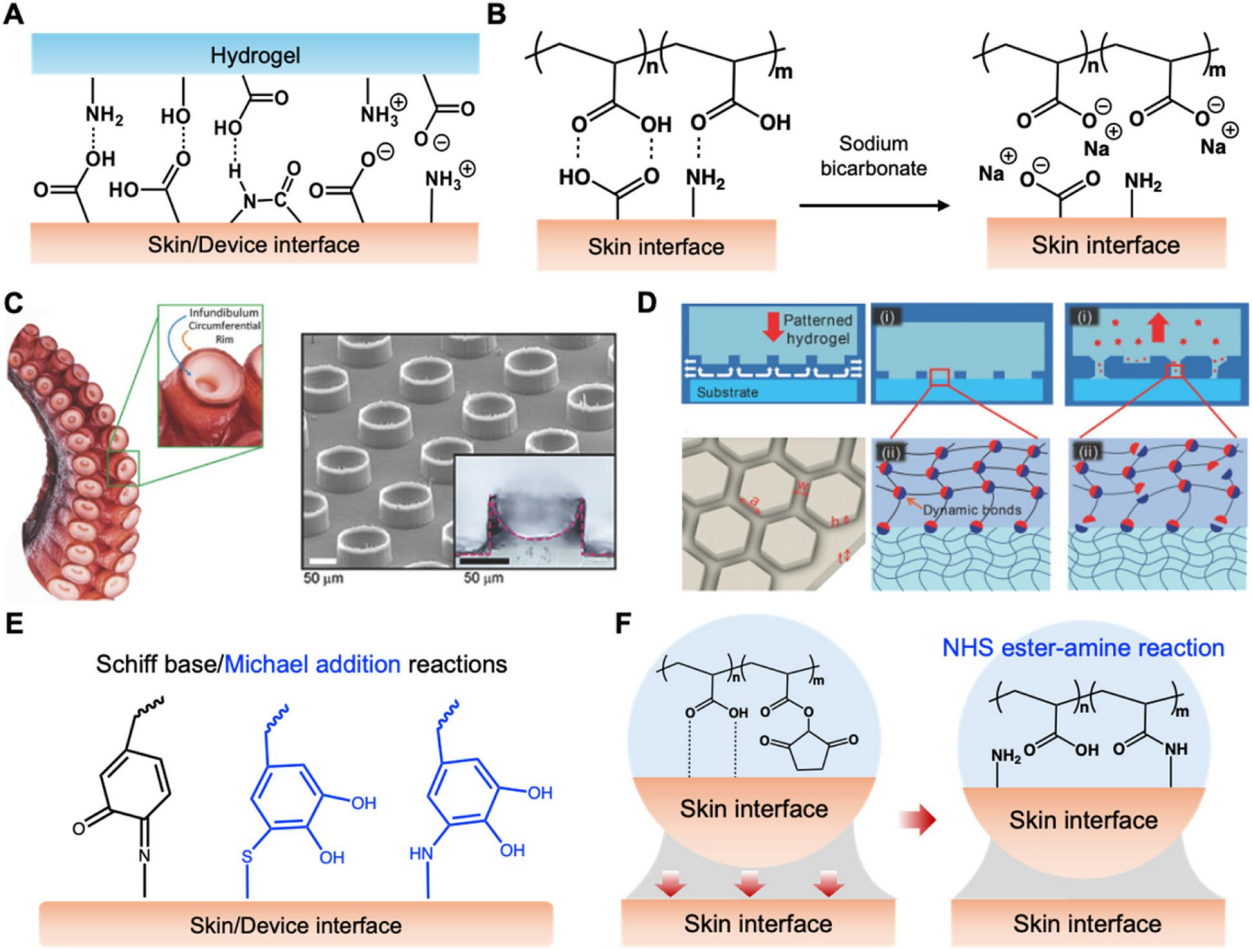
**A** Schematic diagram of adhesion between hydrogel, wearable neural device and skin. **B** Schematic illustrations for the de-cross-linking process of cleavable physical cross-links. **C** SEM image and cross-sectional optical image of artificial microsuckers (100 µm diameter and 75 µm height) inspired by the infundibular and circumferential rim of an octopus sucker [164]. **D** The clingfish-inspired adhesion design features grooves for quick substrate contact, dynamic bonds on hexagonal gel facets dissipate energy during stretching, and independent facets prevent crack propagation, ensuring reversible adhesion [165]. **E** Schematic illustration for conjugation of amine- and thiol of skin interface via Schiff base and Michael addition reaction. **F** The carboxyl groups of double-sided tape form temporary crosslinks with tissue surfaces, and N-Hydroxysuccinimide (NHS) ester groups covalently bond with primary amines on the tissue

Ensuring the safe and stable adhesion of hydrogels to both the skin and the interface of the wearable neurological device is paramount for the device’s consistent functionality [148–152]. Table 2 provides an organized overview of the different adhesion strategies used at the hydrogel-skin and hydrogel-device interfaces, highlighting their respective strengths and limitations, and how they contribute to the overall functionality and user experience of wearable neural devices. Typically, hydrogels incorporate polymer chains with a high density of hydroxyl (-OH), carboxyl (-COOH), or amine (-NH_2_) groups, which can form hydrogen bonds with similar groups on the skin or with the hydrophilic parts of a wearable neural device (Fig. 4A) [153–156]. These bonds are further strengthened when hydrogels are formulated with charged polymers, such as polyanions or polycations, which interact with oppositely charged groups on the skin or device interface (Fig. 4A) [157–161]. Adjusting the pH or ionic strength of the environment can enhance these interactions, providing a tunable adhesion strategy (Fig. 4B). Notably, hydrogels with higher water content tend to exhibit enhanced adhesion due to better conformability to the skin. The increased hydration improves contact between the hydrogel and skin, facilitating the formation of hydrogen bonds with groups like hydroxyl (-OH), carboxyl (-COOH), and amine (-NH_2_). However, if the water content is too high, it can compromise the mechanical integrity of the hydrogel, making it prone to slippage or reducing its long-term adhesion [162]. Conversely, hydrogels with lower water content may exhibit reduced flexibility, limiting their ability to form hydrogen bonds effectively with the skin. While this might improve the structural stability, it could result in weaker interfacial adhesion due to reduced interaction with the skin surface. Additionally, designing hydrogels with nano- or micro-structured surfaces that mimic gecko, octopus, tree frog-inspired adhesion mechanisms, a greater surface area contact to skin interface is achieved, leading to improved adhesion (Fig. 4C and D) [163]. Techniques like nano imprinting or 3D printing can be employed to create these structured surfaces.

**Table 2:** Summary of hydrogel adhesion strategies in wearable neural interfaces

Interface	Adhesion Strategy	Advantages	Disadvantages
Hydrogel-skin interface	Physical interactions (electrostatic, hydrogen bonds)	Easy to removal without damage. Can be tuned for personalized comfort	Less durable than chemical bonds May require reapplication for optimal adhesion
Hydrogel-device interface	Chemical interactions (Schiff base/Michael addition, EDC/NHS coupling)	Creates a strong, durable bond, ensuring device reliability	Complex manufacturing processes More difficult to adjust or remove without potential damage
Enhancemets in adhesion	pH or ionic strength adjustments, nano-or micro-structured surfaces	Adhesion strength is tunable Adaptable to dynamic physiological conditions	Requires advanced fabrication techniques like 3D printing; Higher production costs
Innovation and Personalization	Machine learning optimizations, smart hydrogel functionalities	Personalized device functionality for user comfort and effectiveness	Technological and data integration challenges. Increased complexity and cost in development

In developing WINs, it is advisable to differentiate the adhesion strategies between the hydrogel-skin interface and the hydrogel-device interface. For the attachment of hydrogels to the skin for short time, physical interactions are recommended. This method leverages forces such as van der Waals interactions, electrostatic interactions, and hydrogen bond adhesion, which provide sufficient adhesion to keep the hydrogel in place during use, yet are mild enough to allow easy removal without damaging the skin. On the other hand, the bond between hydrogels and the surfaces of wearable neural devices should ideally be formed through chemical interactions. Utilizing chemical bonding techniques, including in situ Schiff base/Michael addition reactions (Fig. 4E) [166–170], 1-ethyl-3-(3-dimethylaminopropyl)carbodiimide (EDC)/NHS coupling (Fig. 4F) [156, 171–174], and C-H insertion [175–177], ensures a strong and durable connection. These chemical bonds significantly enhance the performance and reliability of the device by ensuring that the hydrogel remains securely attached throughout the device’s usage. In addition, innovations may include hydrogels that change their adhesion strength, conductivity, or drug release profiles in response to the user’s physiological conditions. Furthermore, leveraging machine learning algorithms to predict and optimize the interaction between hydrogels and skin/device interfaces based on user-specific data could personalize the functionality of these devices, increasing both comfort and effectiveness.

## Application of hydrogels in wearable neural interfaces

### Neural recording

Neural recording involves measuring the electrical or biochemical signaling activities of neurons within the brain or broader nervous system. This method is instrumental in investigating brain functions, diagnosing neurological disorders, and designing neuroprosthetic devices. One common neural recording technique is EEG, a non-invasive test that uses electrodes placed on the scalp to record the brain’s electrical activity. Traditional EEG electrodes are categorized into dry, wet, and semi-dry types. Dry electrodes, made of inert metal or Ag/AgCl, do not require conductive gels, reducing preparation time and skin irritation, which makes them suitable for prolonged use [24, 29, 178, 179]. However, they tend to have higher impedance and are prone to motion artifacts, potentially degrading signal quality [24, 28, 30, 180, 181]. Wet electrodes use a commercial conductive gel to enhance skin contact, offering higher signal quality and stable detection. However, these electrodes require skin preparation, which can be time-consuming, uncomfortable, and even cause allergic reactions. Additionally, the conductive gel can dry out, affecting signal integrity during long sessions [24, 29, 179, 182, 183]. Semi-dry electrodes combine the benefits of both dry and wet electrodes by using minimal electrolytes in a porous material to improve signal quality while reducing discomfort [184–188]. These electrodes typically use saline solutions for superior conductivity but managing the release rate of electrolytes to avoid skin irritation or short circuits remains a challenge [189–191].

**Fig. 5:**
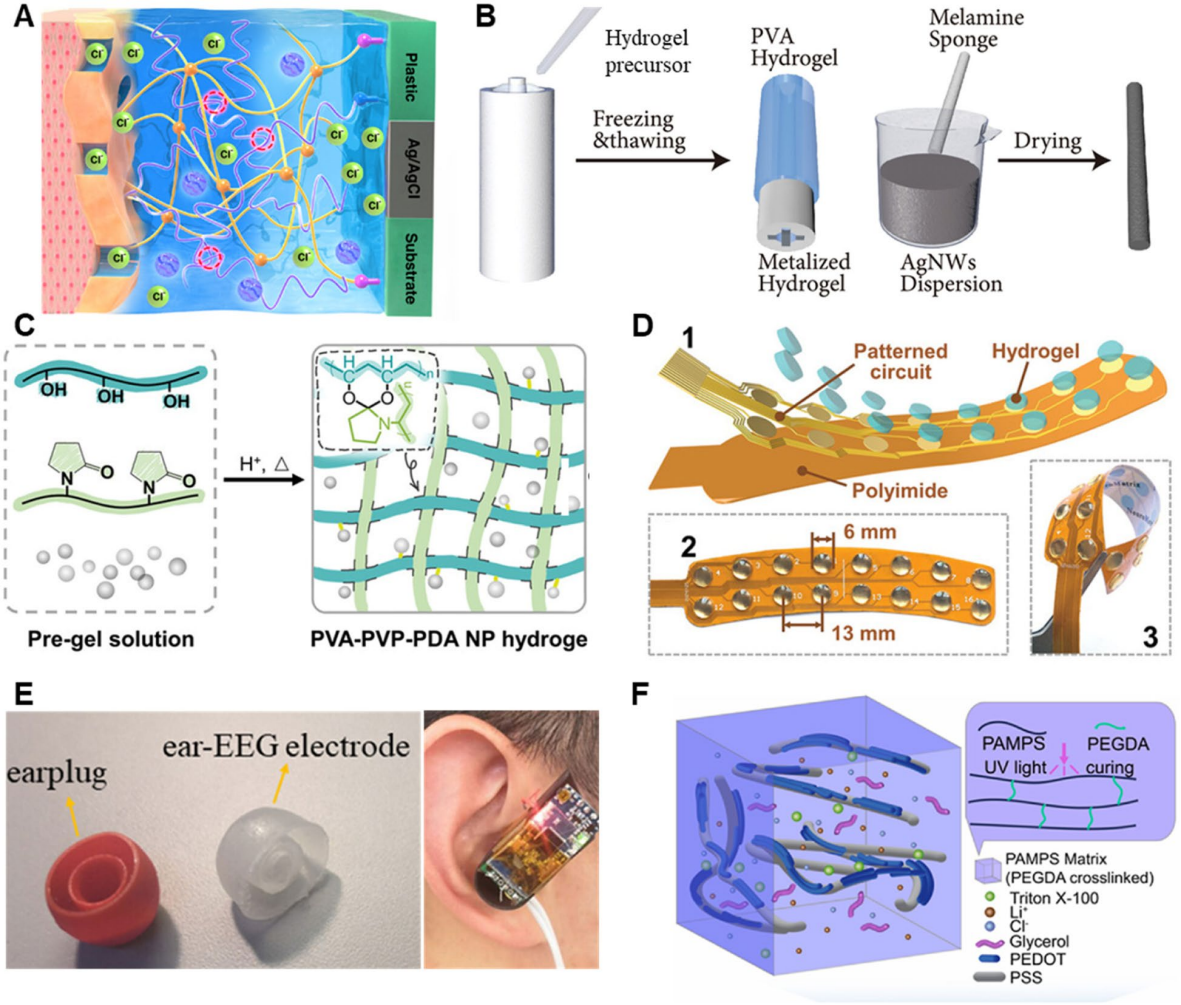
**A** The double-layer hydrogel features a conductive layer for bioelectric signal acquisition, an adhesive layer, supports, and Ag/AgCl assembly [194]. **B** Illustration of the structure of the AgPHMS semidry electrode [195]. **C** Preparation process of PVA-PVP hydrogels with PDA NPs [196]. **D** Schematic illustration and photographs of the multichannel electrodes based on the PVA-PVP-PDA NP hydrogels [196]. **E** Comparison between the self-made ear-EEG electrode earplug (right) and a commercial earphone earplug (left) and the wearing diagram of the wireless ear-EEG system [197]. **F** The schematic diagram of the POLiTAG electrode matrix [86]

In recent decades, hydrogel-based semi-dry electrodes have mitigated many of the challenges faced by their traditional counterparts by enhancing user comfort, maintaining consistent electrolyte levels, and providing stable and high-quality signal transmission. Li et al. developed a series of PVA/polyacrylamide hydrogel electrodes by using physical/chemical crosslinking [192], in-situ polymerization [182], and cyclic freeze-thaw [193] strategy approaches to record EEG on hairy scalps. These electrodes function as an electrolyte reservoir allows for the consistent release of small amounts of saline, minimizing potential drift and reducing electrode-scalp impedance. Xue et al. [194] reported a semidry double-layer hydrogel electrode composed of a high-conductivity conductive layer and a stabilizing adhesive layer. The conductive layer integrates electrolytes and a PEDOT: PSS backbone within a sodium alginate and polyacrylamide network to improve ion mobility and electron conduction. In contrast, the adhesive layer, which contains less water and no electrolytes, gains increased toughness through UV and thermal curing. Both layers, made from acrylamide and alginate, form a cohesive network that captures EEG signals comparably to wet and dry electrodes. However, the complexity of the fabrication process and the specialized materials required make this method challenging to scale up for mass production (Fig. 5A). Liu et al. [195] reported a cost-effective, scalable method to create silver nanowire/triamine sponge hydrogel electrodes. These electrodes continuously deliver electrolytes to the scalp-electrode interface through PVA hydrogel, maintaining low impedance levels between 5–15 kΩ for over 10 h and avoiding short circuits. The study successfully integrated these electrodes into an eight-channel brain–computer interface system for a mental control typing experiment using motion visual evoked potentials (Fig. 5B). However, there are some drawbacks and limitations to consider. The hydrogel in electrodes can dry out over extended use, increasing contact resistance and leading to signal fluctuations, thus requiring regular rehydration or replacement.

To achieve lower impedance and higher precision signals, which are essential for accurate neuro-monitoring, several hydrogel electrodes have been developed to enhance the quality and reliability of neural activity recordings. Han et al. [196] reported an oxidative degradation method to prepare nanosized and transparent polydopamine nanoparticles. These nanoparticles were uniformly integrated within a polyvinyl alcohol/polyvinylpyrrolidone (PVA/PVP) hydrogel through an esterification reaction, enhancing the hydrogel’s self-adhesiveness, conductivity, transparency, and biocompatibility. The resulting multichannel wearable hydrogel electrode established a conformal and stable interface with the skin, demonstrating low interfacial contact impedance (1–100 Hz: 3–4 kΩ), with excellent channel uniformity. Notably, the system accurately analyzed prefrontal EEG signals to assess sustained attention levels, achieving 91.5% accuracy in seven-level classification using a linear support vector machine classifier, significantly outperforming commercial gel electrodes (Fig. 5C and D). Ge et al. [197] developed an ionic conducting hydrogel optimized with 4 wt% hydroxypropyl methylcellulose (HPMC) and 18 wt% PVA. When immersed in a 5 M NaCl solution, the hydrogel demonstrated remarkable conductivity (7.26 S/m) and impressively low impedance (1000 Hz: 8 Ω). The hydrogen bond interactions within the hydrogel increase the distance between cross-links, expanding its microporous structure and enhancing ion flow, thus improving conductivity. Furthermore, these hydrogels were effectively used as ear EEG electrodes in a comprehensive system designed to collect ear EEG data, extract attention feature values, and visually display attention levels via LED color changes (Fig. 5E). Our previous research [86] designed a POLiTAG hydrogel integrating PEDOT: PSS with ionic poly (2-acrylamido-2-methyl-1-propanesulfonic acid), which not only achieves low skin contact resistance (20.7 kΩ cm^2^) but also maintained this low impedance over four weeks (Fig. 5F). Additionally, the utility of POLiTAG electrodes was showcased in various BCI applications, including detecting motor imagery rhythms, error-related potentials, and their integration into a single-channel EEG-based BCI system coupled with functional electrical stimulation for motor rehabilitation.

Another significant concern is the potential for hydrogel drying and dehydration, which can compromise the performance of devices over time. To address this issue, researchers frequently integrate hydrating compounds into the hydrogel formulation [86, 198–200]. As mentioned in Sect. " Conductivity", glycerol is utilized in EEG hydrogels primarily as a water-retaining agent and humidifier. Its hygroscopic properties enable it to absorb and retain water vapor from the surrounding air. This capability stems from the three hydroxyl (-OH) groups in glycerol, which form hydrogen bonds with water molecules, thereby trapping water within the hydrogel matrix. Such hydration is crucial for maintaining the hydrogel’s physical stability and structure, which ensures consistent skin contact for reliable EEG signal acquisition. Moreover, glycerol can alter the rheological properties of the hydrogel, enhancing its adhesion to the skin and negating the need for additional adhesives. It also imparts anti-freezing properties, which are advantageous for the storage and transport of these hydrogels and is economically viable due to its widespread availability. However, high glycerol concentrations may cause skin irritation, particularly with prolonged exposure. Additionally, its hygroscopic nature, while beneficial for maintaining hydration, can lead to excessive moisture absorption in humid environments, potentially altering the hydrogel’s properties. Therefore, optimizing glycerol concentrations and formulations is essential to maximize the performance of hydrogels while minimizing potential drawbacks.

**Fig. 6:**
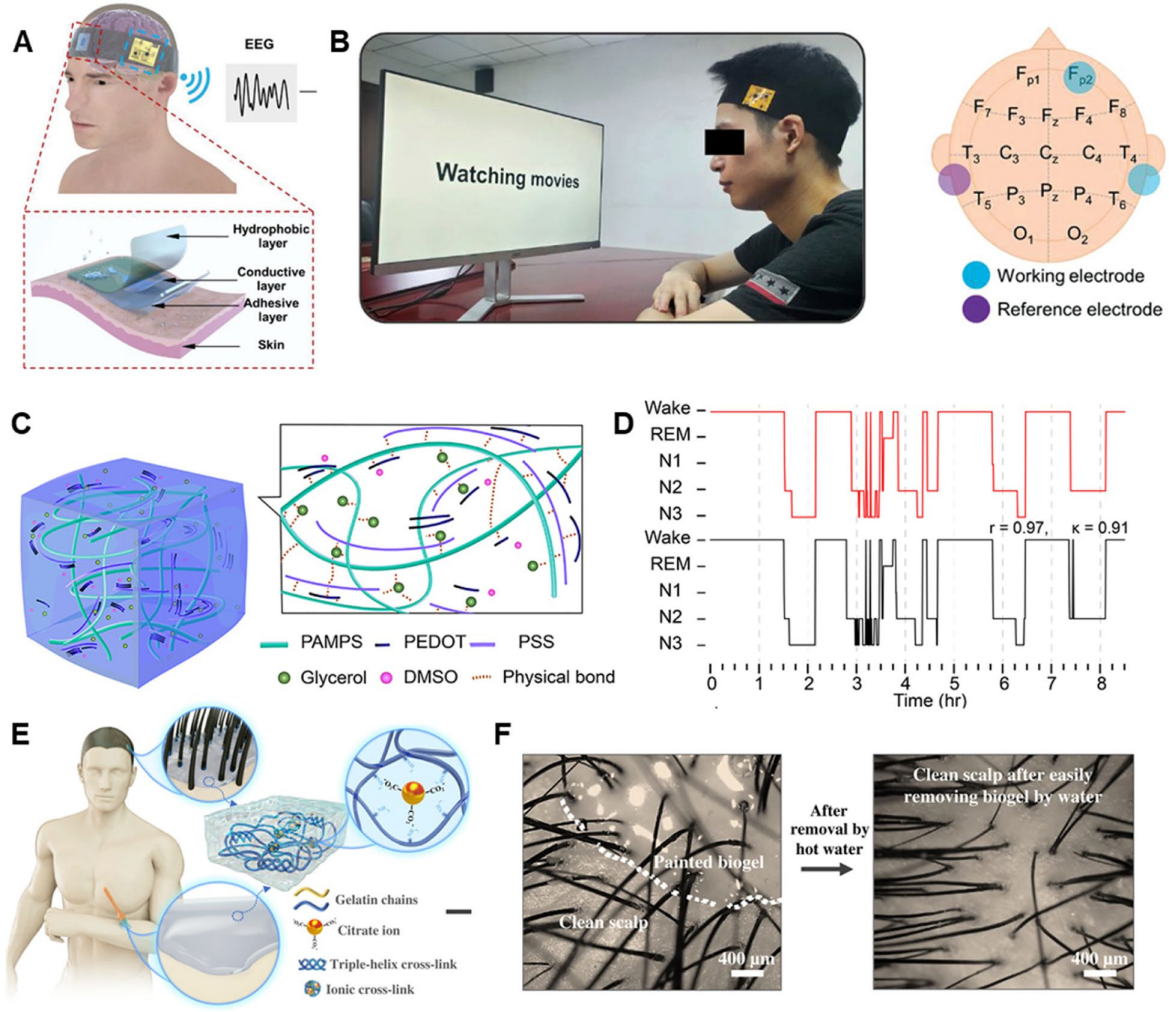
**A** Schematic illustration of AHBH biosensor system for EEG monitoring [201]. **B** A subject watches movie clips for emotion elicitation during EEG monitoring, with a schematic showing Fp2 site, two working electrodes, and a reference electrode on a head model [201]. **C** Schematic of the AIRTrode matrix [52]. **D** A set of representative hypnograms from one of the participants, showing the sleep stage classifications with the data recorded from both the AIRTrode and the commercial EEG gel [52]. **E** Schematic of paintable biogel for EEG on hairy scalp [202]. **F** optical images show biogel application and easy removal, ensuring good contact [202]

In addition to incorporating hydrating compounds, the design of dual-layer hydrogels featuring both hydrophilic and hydrophobic layers offers a promising method to prevent dehydration and drying of hydrogel in long-term wearable neural devices. The hydrophilic layer, with its affinity for water, is crucial for maintaining hydration within the hydrogel. This ensures a continuous supply of moisture necessary for maintaining conductivity, which is essential for accurate signal transmission. In contrast, the hydrophobic layer acts as an environmental barrier, repelling water to seal in moisture, thereby extending the device’s operational lifespan and reducing the need for frequent maintenance or replacements. These layers work in concert to significantly enhance both the durability and functionality of hydrogel in wearable neural devices. For example, Yang et al. [201] developed on-skin biosensors with adhesive and hydrophobic bilayer hydrogels that simultaneously possess high adhesion (59.7 N m^−1^) and hydrophobicity (133.87°) as device/skin interfaces to achieve high-fidelity measurements of evoked potential signals and classification of human emotion (Fig. 6A and B).

It should be noted that when hydrogel electrodes are used on subjects with dense hair, the presence of hair can significantly obstruct the electrode’s ability to maintain direct contact with the scalp. This complicates the setup and potentially affects the signal quality. Recently, our research group developed an adhesive, injectable, room-temperature spontaneously cross-linked (AIRTrode) hydrogel electrode for prolonged EEG monitoring, proving particularly effective on hairy scalp regions (Fig. 6C) [52]. With an impedance of only 17.53 kΩ during recordings exceeding 8 h, AIRTrode offered a significantly higher signal-to-noise ratio (23.97 dB) compared to traditional commercial wet electrodes (17.98 dB). In overnight sleep EEG monitoring tests, AIRTrode demonstrated outstanding stability and maintained high signal quality over 8 h, showing a high correlation in sleep stage classification when compared to commercial electrodes, with a correlation coefficient (r) of 0.91 and Cohen’s kappa (κ) of 0.84 (Fig. 6 D). This suggests a strong potential for AIRTrode to enhance EEG monitoring in challenging scenarios. Although injectable hydrogel electrodes are feasible for use on hairy scalp surfaces, there is limited attention given to the removal of hydrogel residues from hair after testing. This oversight can lead to user discomfort and challenges in post-experiment cleanup. One possible solution could be the development of hydrogels with reversible adhesive properties that can be easily deactivated, allowing the hydrogel to be removed cleanly without residue. For example, Wang et al. [202] developed a gelatin-based biocompatible on-skin ionic conductive hydrogel capable of temperature-controlled reversible phase transitions between a fluidic state and a viscoelastic gel state, alongside water-triggered removal properties. The hydrogel’s dynamic properties enable it to achieve conformal contact with the hairy scalp, ensuring high-fidelity EEG recording without the need for additional mechanical fixtures to hold the electrodes in place (Fig. 6E). This hydrogel can effectively capture steady-state visually evoked potentials, with high classification accuracy facilitated by a convolutional neural network-based learning architecture. Another approach involves formulating the hydrogel with substances that dissolve or degrade safely in a biocompatible solvent, which can be applied post-testing to facilitate easy removal (Fig. 6F).

### Neurostimulation

Neurostimulation is a medical technique that employs electrical signals or other forms of energy to directly stimulate parts of the nervous system. It is commonly used to manage chronic pain [203, 204], neurological disorders [205–207], and some psychiatric conditions [208, 209] by modulating the activity of nerve cells. Hydrogels play a critical role as the interface between neurostimulation devices and the patient’s body, efficiently transmitting therapeutic signals generated by the device to the targeted nerve [45, 210–212]. Additionally, some hydrogel electrodes can be engineered to combine drug delivery and stimulation functions by releasing therapeutic agents directly at the site of stimulation to enhance treatment outcomes [213–215]. Currently, implantable neural devices are the predominant application of hydrogels in neurostimulation [38, 216–220]. Table 3 provides a comparative summary between WNIs and traditional implantable neural interfaces, highlighting the relative strengths and weaknesses of each approach, offering clarity for informed decision-making in their application and development. However, in this review, our primary focus is on their non-invasive technology in wearable neural devices. Thus, the following section of our review aims to comprehensively detail wearable neurostimulation technologies and discuss both the roles and challenges of hydrogels in these applications.

**Table 3:** Comparison of WNIs and implantable neural interfaces

Aspect	Wearable Neural Interfaces (WNIs)	Implantable Neural Interfaces
Signal resolution and depth	Surface neural activity, ideal for daily use, Enhanced depth targeting (WNIs with FUS)	High resolution but requires surgery for deep targets
Susceptibility to signal interference	Optimized design reduces interference, lightweight and easy to use	Stable, but complex surgery and long-term risks
Reliability of power supply	External power, easy recharging, no surgery needed	Requires surgery for battery replacement, more complex management
Data security	Wireless, secured by encryption	Also vulnerable to wireless risks, surgery needed for upgrades
Long-term durability	Replaceable components, easy to maintain	Long-term use affected by immune response and degradation

**Fig. 7:**
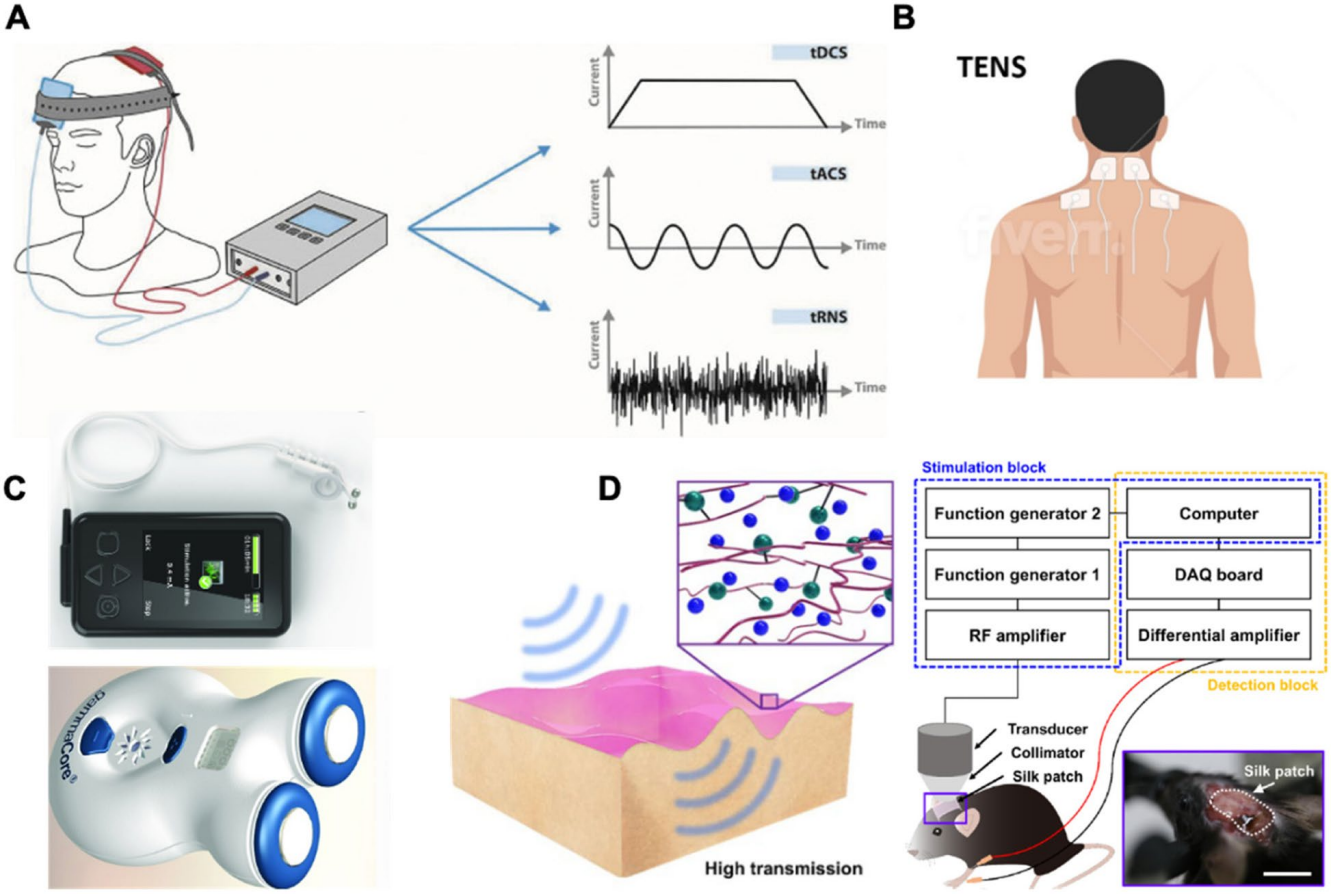
**A** TES uses weak electrical currents applied to the head for 5–30 min via electrodes, modulating neuronal activity through direct (tDCS), random noise (tRNS), or alternating (tACS) stimulation [224]. **B** TENS is applied to nerve dermatomes or areas of muscle pain [228]. **C** Non-implantable commerical VNS systems: (a) NEMOS (top) and (b) gammaCore (bottom) [229]. **D** Conceptual illustrations of the Ca-modified silk patch before and after hydration and their application for the brain stimulation setup (scale bar: 8 mm) [230]

Transcranial electrical stimulation (TES) is an emerging non-invasive neurostimulation technique that includes transcranial direct current stimulation (tDCS), transcranial alternating current stimulation (tACS), and transcranial pulsed current stimulation (tPCS) [53, 221]. TES involves placing two or more extracranial hydrogel electrodes on the scalp/skull to generate a current entering the brain via the potential difference between the electrodes (Fig. 7A) [222–224]. This technology has been shown to enhance verbal recognition memory tasks in patients with Alzheimer’s disease and to promote and restore functional balance in stroke patients. In rodent models, TES hydrogel electrodes are placed on the surface of the skull to facilitate safe, long-term experimentation. The results from these models can be extrapolated to human subjects, where the placement of TES electrodes on the scalp is influenced by the relative ohmic conductivity properties of subcutaneous tissues [211, 225, 226]. In addition, TES hydrogel electrodes typically require larger surface areas (20–35 cm^2^) to reduce the electrochemical impedance at the electrode-tissue interface [211, 227]. However, this large surface area may affect their capability to provide localized stimulation for high spatial resolution neural modulation. Although smaller electrodes (about 1 cm^2^) have been reported to establish more focal stimulation, multiple electrodes are required to influence neural circuits. It should be noted that non-invasive hydrogel electrodes have limitations in stimulation precision and depth, which may impact the specificity and efficacy of the therapy. Ideally, TES hydrogel electrodes should be comfortable, flexible, biocompatible, and stable in physiological environments, possess high charge capacity for effective signal interaction, and provide mechanical support to maintain proper contact with the skull or tissue.

Transcutaneous electrical nerve stimulation (TENS) is a therapeutic technique used for pain management [231–233]. It involves the use of a small, battery-operated device that delivers low-voltage electrical currents through hydrogel electrodes attached to the skin [234]. The intensity and frequency of the electrical impulses can be adjusted depending on the user’s needs and the specific condition being treated [235, 236]. TENS is commonly used for chronic musculoskeletal pain, such as back pain or arthritis, as well as for acute pain, like that after surgery or trauma (Fig. 7B) [228, 237–240]. The effectiveness of hydrogel in TENS depends on its ability to maintain good electrical contact between the electrodes and the skin. Moreover, hydrogel’s performance can vary based on environmental factors like temperature and humidity, which can affect its conductivity. Therefore, developing hydrogels that can maintain their properties under different environmental conditions (temperature and humidity) and during movement remains a technical challenge.

Vagus nerve stimulation (VNS) is primarily utilized for treating conditions such as epilepsy and depression [241–244]. Traditionally, VNS involves a surgical procedure to implant a small pulse generator under the skin in the chest area, connected via a lead wire that encircles the vagus nerve in the neck [241, 245, 246]. This setup allows for the continuous delivery of electrical signals, which can be adjusted by healthcare providers to optimize therapeutic outcomes. However, the invasiveness of the procedure and the need for periodic battery replacements are significant considerations for potential recipients. In contrast, newly developed non-invasive VNS devices offer a less intrusive alternative (Fig. 7C) [229, 247–251]. These external devices use hydrogel electrodes applied to the skin, typically near the ear where the auricular branch of the vagus nerve is accessible externally. This non-surgical approach is safer and more accessible, avoiding the complications associated with invasive procedures. However, ensuring that non-invasive VNS matches the efficacy of traditional implanted devices remains a challenge. The external hydrogel electrodes must effectively stimulate the vagus nerve through the skin, a task that may not replicate the direct stimulation achieved by implanted leads. Moreover, finding the optimal placement for these electrodes to consistently target the correct nerve fibers is crucial yet challenging, with incorrect positioning potentially diminishing the therapy’s effectiveness and leading to inconsistent results.

Focused ultrasound (FUS) is a recently emerging non-invasive neurostimulation technique that utilizes precisely controlled high-intensity sound waves to target specific neural structures non-invasively through the skin [252–257]. These waves can penetrate the skull and other body tissues without incisions, reaching deep-seated neural structures [258, 259]. The focus and intensity of the ultrasound waves can be precisely controlled to stimulate or suppress neural activity in targeted regions. The ultrasound waves induce mechanical and thermal effects at the focal point, which can alter the electrical properties of neural tissues [252, 255]. For instance, it has been explored for its potential to disrupt faulty neural circuits in Parkinson’s disease [260–262], alleviate symptoms of essential tremors [263–265], and manage chronic pain [266–268]. Hydrogels play an essential role in FUS for neural stimulation by serving as an acoustic coupling agent [269–271]. Thanks to their high-water content and excellent acoustic conductivity, hydrogels enhance the efficiency of ultrasound wave transmission in wearable devices. These properties allow hydrogels to distribute ultrasound waves more uniformly and minimize air gaps between the skin and the device, crucial for improving energy transmission [272]. Lee et al. proposed a wearable ultrasound patch composed of Ca-modified silk, providing a stable interface, coupling medium, flexible transducer array, and miniaturized circuitry. The patch ensures high adhesion, low ultrasound transmission loss, and performs comparably to commercial ultrasound gel (Fig. 7D) [230]. However, challenges such as decreased adhesion over long-term wear can cause skin irritation or allergic reactions, particularly in users with sensitive skin. To overcome the drawbacks associated with conventional ultrasound gels, our group recently designed a bioadhesive hydrogel that ensures long-term stability as an acoustic couplant. This hydrogel exhibited less than 13% reduction in acoustic intensity and maintained a consistent adhesion force of 0.961 N/cm for up to 35 days. By integrating this hydrogel with our developed wearable miniaturized ultrasound device, we were able to effectively suppress somatosensory evoked potentials triggered by median nerve stimulation through functional electrical stimulation, sustaining this effect over a 28-day period [272]. Nevertheless, researchers still need to further improve and develop new hydrogel materials that not only possess strong adhesion but also offer greater durability and enhanced biocompatibility. Additionally, hydrogels offer therapeutic benefits during ultrasound treatments. Their cooling properties can protect the skin from thermal damage due to prolonged exposure to high-energy ultrasound waves, while their soft texture increases patient comfort, making them particularly beneficial in treatments that require long durations or repeated sessions.

## Perspectives

### Design of hydrogel electrodes on hairy skin

Electrophysiological signal monitoring using hydrogel electrodes on hairy skin presents a series of unique challenges, particularly for WNIs, where maintaining adhesion and stability is crucial. This complexity arises from the need to navigate through hair, which can obstruct consistent electrode contact and thus impair the functionality and reliability of the readings. One possible solution is to develop condition-responsive hydrogels. Those hydrogel offers a novel solution to these adhesion challenges. For instance, a temperature-responsive hydrogel exhibits enhanced adhesive capabilities at body temperature, ensuring a secure contact quality during use. Conversely, they can be easily removed at lower temperatures, thereby minimizing discomfort and potential damage to skin or hair. Furthermore, the design of hydrogel electrodes must be optimized between adhesion and mechanical properties to effectively penetrate or navigate through the hair, allowing them to conform to the uneven surface of hairy skin without tearing or detaching.

### Long-term wearable neural interfaces

Hydrogels used in WNIs should maintain their stability to various environmental factors such as heat, humidity, skin oils, and sweat. Moreover, hydrogels in long-term WNIs should be designed to minimize dehydration. Techniques such as embedding superabsorbent polymers could help retain moisture within the hydrogel structure. Furthermore, future hydrogel designs should improve customization to fit individual anatomical and physiological differences, enhancing comfort and therapeutic efficacy. For example, hydrogels for FUS devices could incorporate characteristics such as adjustable thickness, elasticity, and permeability to ultrasound waves. Additionally, integrating sensor technologies with hydrogels in WNIs could allow real-time monitoring of vitality conditions and therapy efficacy. Sensors embedded within or alongside the hydrogel could track changes in skin temperature, hydration levels, and ultrasound transmission quality, providing feedback for dynamically adjusting therapy parameters.

### Integration of hydrogels with electronic components

The integration of hydrogels with electronic components for wearable neural devices represents a cutting-edge development in both the fields of material science and neurotechnology. This combination is poised to transform how neural devices are designed, enhancing their functionality and user experience, particularly in monitoring and stimulating neural activity. For neurological diseases, prevention is often focused on monitoring and early intervention. Wearable neural devices equipped with hydrogel-electronic interfaces can continuously monitor neurological activity and physiological parameters, identifying abnormal patterns or changes that may precede a neurological event. For example, such devices can detect early signs of seizure activity, allowing for immediate intervention through neuromodulation to prevent the onset of a full-blown epileptic seizure. Similarly, early detection of the precursors to migraine attacks can enable preemptive administration of neuromodulatory therapies to mitigate or even prevent the onset of symptoms. In rehabilitation, hydrogel-based wearable devices focus on restoring function and reducing disability by facilitating and enhancing the neurorecovery process. For stroke recovery, targeted electrical stimulation helps retrain and strengthen muscles and neural pathways, improving motor function and accelerating recovery. For Parkinson’s disease, continuous neuromodulation can help manage symptoms such as tremors and rigidity, improving patient mobility and quality of life.

## Conclusion

In this review, we examined the crucial role of hydrogels in wearable neural interfaces, highlighting their essential contributions to improving the connection between electronic devices and neural tissues. We then discussed recent advancements in hydrogel technology that had enhanced signal fidelity and stability for neural recording and stimulation. The review also outlined future research directions, including the development of hydrogel electrodes suited for hairy skin, long-term wearable neural interfaces, and the integration of hydrogels with electronic components. This analysis emphasized the transformative potential of hydrogels in augmenting the functionality and user-friendliness of wearable neural interfaces, setting the stage for their broader adoption in both medical and consumer electronics.

## Data Availability

Not applicable.
